# Vigeo Promotes Myotube Differentiation and Protects Dexamethasone-Induced Skeletal Muscle Atrophy via Regulating the Protein Degradation, AKT/mTOR, and AMPK/Sirt-1/PGC1α Signaling Pathway In Vitro and In Vivo

**DOI:** 10.3390/nu16162687

**Published:** 2024-08-13

**Authors:** Yoon-Hee Cheon, Chang-Hoon Lee, Chong-Hyuk Chung, Ju-Young Kim, Myeung-Su Lee

**Affiliations:** 1 Musculoskeletal and Immune Disease Research Institute, School of Medicine, Wonkwang University, 460 Iksandae-ro, Iksan 54538, Republic of Korea; hanleuni@naver.com (Y.-H.C.); lch110@wku.ac.kr (C.-H.L.); taylorchung@hanmail.net (C.-H.C.); 2 Division of Rheumatology, Department of Internal Medicine, Wonkwang University Hospital, 460 Iksandae-ro, Iksan 54538, Republic of Korea

**Keywords:** Vigeo, nuruk fermentation, muscle atrophy, sarcopenia, dexamethasone

## Abstract

Sarcopenia, a condition caused by an imbalance between muscle growth and loss, can severely affect the quality of life of elderly patients with metabolic, inflammatory, and cancer diseases. Vigeo, a nuruk-fermented extract of three plants (*Eleutherococcus senticosus* Maxim (ESM), *Achyranthes japonica* (Miq.) Nakai (AJN), and *Atractylodes japonica* Koidzumi (AJK)) has been reported to have anti-osteoporotic effects. However, evidence of the effects of Vigeo on muscle atrophy is not available. Here, in the in vivo model of dexamethasone (Dex)-induced muscle atrophy, Vigeo treatment significantly reversed Dex-induced decreases in calf muscle volume, gastrocnemius (GA) muscle weight, and histological cross-section area. In addition, in mRNA and protein analyses isolated from GA muscle, we observed that Vigeo significantly protected against Dex-induced mouse muscle atrophy by inhibiting protein degradation regulated by atrogin and MuRF-1. Moreover, we demonstrated that Vigeo significantly promoted C2C12 cell line differentiation, as evidenced by the increased width and length of myotubes, and the increased number of fused myotubes with three or more nuclei. Vigeo alleviated the formation of myotubes compared to the control group. Vigeo also significantly increased the mRNA and protein expression of myosin heavy chain (MyHC), MyoD, and myogenin compared to that in the control. Vigeo treatment significantly reduced the mRNA and protein expression of muscle degradation markers atrogin-1 and muscle RING Finger 1 (MuRF-1) in the C2C12 cell line in vitro. Vigeo also activated the AMP-activated protein kinase (AMPK)/silent information regulator 1 (Sirt-1)/peroxisome proliferator-activated receptor-γ co-activator-1α (PGC1α) mitochondrial biogenesis pathway and the Akt/mTOR protein synthesis signaling pathway in Dex-induced myotube atrophy. These findings suggest that Vigeo may have protective effects against Dex-induced muscle atrophy. Therefore, we propose Vigeo as a supplement or potential therapeutic agent to prevent or treat sarcopenia accompanied by muscle atrophy and degeneration.

## 1. Introduction

Muscles are specialized organs that enable physical movement and function, including vital actions, such as chewing and breathing [1]. Especially for older adults, maintaining adequate muscle mass is one of the most important components of preventing metabolic disorders and maintaining a healthy life in old age. Muscle loss is a common feature of several muscle-related diseases, including muscle atrophy, muscular dystrophy [2], sarcopenia [3], and cachexia [4]. Sarcopenia is one of the most important age-related anatomical changes that is associated with decreased skeletal muscle mass and function. In addition to aging, other external factors that contribute to sarcopenia include physical inactivity, malnutrition, stress, and environmental factors. Age-related molecular changes that can cause sarcopenia include changes in transcription factors, structural proteins, and signaling molecules [5]. Several transcription factors are involved in muscle cell differentiation, including MyoD, Myf5, myogenin, and myogenic regulatory factor4 (MRF4) [6]. These transcription factors promote the expression of genes that are essential for muscle cell development. Myosin heavy chain (MyHC) and sarcomeric α-actinin are myotube-specific structural proteins regulated by transcription factors such as MyoD and myogenin [7]. Signaling molecules are proteins that transmit signals from one cell to another. Signaling events involved in the control of skeletal muscle atrophy and hypertrophy are also important for maintaining healthy skeletal muscle conditions [8].

Preclinical sarcopenia research is primarily performed using animal models of atrophy and in vitro models. The dexamethasone (Dex)-induced sarcopenia model is a common chemically induced atrophy experimental tool in mice. Dex is a synthetic glucocorticoid that induces the loss of muscle mass and muscle weakness. Muscle atrophy induced by Dex is accompanied by the suppression of protein synthesis and the induction of protein catabolism, including two muscle-specific E3 ubiquitin ligases, muscle atrophy F-box (MAFbx, also known as atrogin-1) and muscle RING Finger 1 (MuRF-1). Numerous studies have used atrogin-1 and MuRF-1 to investigate diseases based on their functional roles and the regulation of muscle lytic factors in muscle atrophy [9].

In recent years, a growing body of evidence has shown that various natural and fermented plant extracts have beneficial effects on muscle cell differentiation and function. For example, Yun et al. (2021) found that *Aronia melanocarpa* extract improved muscle cell growth and differentiation and attenuated Dex-induced muscle atrophy in vivo [10]. Dex is a glucocorticoid widely used to induce muscle atrophy in vivo by increasing proteolysis. As an example of an experiment using an in vivo model of Dex-induced mouse muscle atrophy, a study by Lee et al. (2023) has shown that the extract of *Alnus japonica* improved muscle strength and mass and inhibited dexamethasone-induced muscle atrophy in mice [11]. In a recently published study from our group, Vigeo, a nuruk ferment combining the three substances *Eleutherococcus senticosus* Maxim (ESM; 135 g), *Achyranthes japonica* Nakai (AJN; 78 g), and *Atractylodes japonica* Koidzymi (AJK; 78 g), has been shown to have inhibitory effects on lipopolysaccharide (LPS)-induced osteoporosis in animal models [12]. However, no evidence is available on its effects on muscle cell differentiation and formation in vivo or in vitro. Therefore, this study aimed to investigate the effects of Vigeo on muscle cell differentiation in a dexamethasone-induced muscle atrophy disease model.

## 2. Materials and Methods

### 2.1. Preparation of Vigeo

Vigeo was purchased from Panax Bio Co., Ltd. (Nonsan, Republic of Korea). Vigeo was prepared using the traditional Korean fermentation method described in detail in our previous study [12]. Briefly, dried ESM (135 g), AJN (78 g), and AJK (78 g) were extracted in hot water for 3 h in a Kyungseo extractor (COSMOS-660; Kyungseo E&P Co., Ltd., Inchoeon, Republic of Korea). Korean traditional fermentation starter, *nuruk* (1 kg), was mixed with fresh yeast (4 g) and distilled water (1.5 L); the mixture was fermented for 96 h. Then, popped rice (3 kg) and a combined extract (hot water extract) were mixed and fermented at 26 °C for 15 days. The nuruk fermented extract mixture (Vigeo) was freeze-dried and then dissolved in distilled water (DW) according to the experimental concentration. All experiments were performed with the same batch of Vigeo, and a patent for the composition of matter and manufacture method has been registered with the Korean Intellectual Property Office (Patent No.: 10-1512230-0000).

### 2.2. Animals

All animal procedures conformed to Korea’s Animal Welfare Legislation with efforts made to minimize the number of animals and their discomfort. All of the animal experiments were conducted under the Approval of Institutional Animal Committee of Wonkwang University (approval no. WKU23-78); 8-week-old male ICR mice (weighing 33–36 g, *n* = 20) were purchased from Samtako (Daejeon, Republic of Korea) and housed in a temperature (22–24 °C) and humidity (55–60%) controlled facility on a 12 h light/dark cycle. All mice were provided ad libitum access to water and a standard irradiated chow diet (SAM #31; Samtako Bio-Korea Inc., Osan, Korea). The experimental mice were randomly assigned to one of the following four groups using a computerized random number generator: control (*n* = 5), Dex (25 mg/kg)-induced (*n* = 5), Dex + Vigeo Low (200 mg/kg, *n* = 5), and Dex + Vigeo High (500 mg/kg, *n* = 5). The staff who assign and manage groups of mice are different from the researchers who evaluate their effectiveness. Mice and tissue samples were labeled to ensure that researchers evaluating treatment effectiveness and analyzing results were not aware of differences among groups. Muscle atrophy was induced in mice by intraperitoneal injection of dexamethasone daily for 8 d [13]. Vigeo and DW were orally administered daily for 8 days. At the end of the experiment, the mice were euthanized via cervical dislocation; gastrocnemius (GA), soleus (SOL), tibialis anterior (TA), and extensor digitorum longus (EDL) muscle were collected for subsequent experiments. All animal studies did not specifically control for potential confounders. Mice were closely monitored throughout the experiment to assess their health status. During the experiment, no experimental units or data points were excluded for any of the animals because there were no significant changes in body weight, responses, or behaviors following drug administration.

### 2.3. C2C12 Cell Line Culture and Differentiation

The C2C12 myoblasts, a mouse myoblast cell line, were purchased from American Type Culture Collection (ATCC, Manassas, VA, USA) and maintained in growth medium (GM), Dulbecco’s modified Eagle’s medium (DMEM, Gibco BRL, Grand Island, NY,USA) with 10% fetal bovine serum (FBS) and 1% penicillin and streptomycin (P/S) antibiotic. C2C12 cells (5 × 10^3^ cells/well) were plated in 48-well plates and were grown to 80–90% confluence for 2–3 days. To induce differentiation of C2C12 cells from myoblasts to myotubes, the growth medium was replaced from GM to differential medium (DM) containing 2% horse serum and further cultured for 5–6 days. The medium was replaced every 3 days. After differentiation, myotubes were further cultured with Dex (100 μM) for 48 h at DM 5 days.

### 2.4. C2C12 Cell Cytotoxicity

Cell viability was measured using a Cell Proliferation Kit II to confirm the potential cytotoxic effects of Vigeo. C2C12 cells (1 × 10^3^ cells/well) were seeded in 96-well plates with various concentrations of Vigeo and incubated for 5 days. Further, 50 μL of XTT reagent was added to each well, followed by another incubation for 40 min. Cell cytotoxicity was determined by measuring the absorbance at 540 nm in four replicates using a multi-well plate reader (Epoch; BioTek Instruments, Winooski, VT, USA).

### 2.5. Quantitative Real-Time PCR (qRT-PCR)

Total RNA from C2C12 myotubes or gastrocnemius muscle was isolated using TRIzol reagent (Thermo Fisher Scientific, Waltham, MA, USA) according to the manufacturer’s instructions. cDNA prepared using the Revertaid first strand cDNA synthesis kit (Thermo Fisher Scientific) was used as a template for qRT-PCR. qRT-PCR was performed to investigate the expression of the following interested genes: musGAPDH for 5′-TCA AGA AGG TGG TGA AGC AG-3′, musGAPDH rev 5′-AGT GGG AGT TGC TGT TGA AGT-3′, musMyoD for 5′-CGC TCC AAC TCT GAT-3′, musMyoD rev 5′-TAG TAG GCG GTG TCG TAG CC-3′, musMyogenin for 5′-CTA CAG GCC TTG CTC AGC T-3′, musMyogenin rev 5′-AGA TTG TGG GCG TCT GTA GG-3′, musMyHC1 for 5′-GCC CAG TGG AGG ACA AAA TA-3′, musMyHC1 rev 5′-TCT ACG TGC TCC TCA GCA T-3′, musAtrogin-1 for5′- CTG GAT TGG AAG AAG ATG TA-3′, musAtrogin-1 rev 5′-CTT GAG GGG AAA GTG AGA CG-3′ musMuRF1 for 5′-CCT ACT TGC TCC TTG TGC-3′, musMuRF1 rev 5′-TCC TGC TCC TGC GTG AT-3′. All data were normalized with GAPDH. The amplification parameters consisted of 40 cycles of an initial denaturation at 95 °C for 15 min, followed by 40 cycles of denaturation at 95 °C for 1 min, annealing at 60 °C for 30 s, and extension at 72 °C for 1 min. The 2^−ΔΔct^ method was used for quantification [14].

### 2.6. Western Blot

C2C12 cells or gastrocnemius muscle tissues were subjected to cold lysis buffer and whole protein lysates were extracted. About 20–30 μg of protein in each well was run by sodium-dodecyl sulfate polyacrylamide gel electrophoresis (SDS-PAGE) 6–15% followed by transfer to polyvinylidene fluoride (PVDF) membranes. The membranes were blocked for 60 min at 37 °C with skim milk of 5% in TBST buffer and the membranes were incubated overnight with the following primary antibodies: anti-MyHC1, anti-MyoD, anti-myogenin, anti-atrogin-1, anti-MuRF-1, anti- silent information regulator 1 (Sirt-1; Santa Cruz Biotechnology, Santa Cruz, CA, USA); anti- peroxisome proliferator-activated receptor-γ co-activator-1α (PGC1α; Proteintech, Rosemont, IL, USA); anti-β-actin (Sigma-Aldrich, St. Rouis, MO, USA); anti-GAPDH, anti-p-ACC, anti-ACC, anti-p-AMP-activated protein kinase (AMPK), anti-AMPK, anti-p-AKT, anti-AKT, anti-p-mTOR, anti-mTOR, anti-p-70-kDa ribosomal S6 protein kinase (p70S6K1), anti-p70S6K1, anti-p-eIF4E-binding protein (4EBP1), and anti-4EBP1 (Cell Signaling Technology, Beverly, MA, USA). Secondary antibodies of anti-rabbit and anti-mouse (Enzo Life Sciences, New York, NY, USA) were incubated for 1 h. Specific protein signals were detected using WestGlow^TM^ FEMTO ECL Chemiluminescent substrate (Biomax, Guri-si, Gyeonggi-do, Republic of Korea).

### 2.7. Immunofluorescence

C2C12 cells of 5 × 10^3^ on 48well were grown in DM for 5 d in the presence or absence of Vigeo and exposed to Dex (100 μM) for 48 h to induce atrophy. The cells were fixed with 3.7% formaldehyde in PBS for 15 min and permeabilized with Tween 20 for 10 min. Myoblasts and myotubes were identified by MyHC1 immunofluorescence staining (Santa Cruz Biotechnology). Nuclei were visualized by blue DAPI staining (Sigma-Aldrich, St. Louis, MO, USA). Secondary antibodies were stained with Alexa Fluor^™^ 488 goat anti-Mouse IgG (H + L) (Invitrogen Life Technologies, Carlsbad, CA, USA) in the dark at room temperature.

### 2.8. Micro-CT (mCT)

The muscle samples were scanned using a mCT scanner (NFR-Polaris-G90; NanoFocusRay, Jeoju, Republic of Korea). Images were acquired at 50 kVp, 55 μA, 6 μm, and 133 mSec, and 720 views were present. The final reconstructed images were converted into a digital imaging and communication-in-medicine (DICOM) file. The mCT data files were quantified using the Xelis 3D-Image calculator software 2.0 (Infinitt, Seoul, Republic of Korea).

### 2.9. Tissue Staining

Gastrocnemius muscle tissues were isolated and fixed with 10% neutral buffered formalin for 1 day. Paraffin sections 5 μm-thick were sliced and stained with hematoxylin and eosin (H&E). After H&E staining, representative images were taken on a Nikon Ts2 microscope (Nikon; Shinagawaku, Tokyo, Japan) for use in the cross-sectional area (CSA) analysis. The CSAs of individual myofibers were measured using NIS Elements version 5.3 software (Nikon), which can quantify the area within myofibers. After all CSA measurements were collected, CSA distribution graphs were generated by binning all data to produce a parametric distribution in control groups and then comparing experimental distributions using the same binning.

### 2.10. Statistical Analysis

The sample size estimation was performed a priori using the G power program (https://clinicalc.com/stats/samplesize.aspx, accessed on 12 September 2023). All experiments were examined at least three times, and the data were expressed as the mean ± Standard deviation. Statistical significance was determined using one-way analysis of variance (ANOVA), followed by Tukey’s multiple comparison test and Student’s *t*-test. Differences were considered statistically significant at *p* < 0.05. Residual diagnostics were conducted to check the normality and linearity assumptions.

## 3. Results

### 3.1. Vigeo Ameliorates Muscle Wasting in a Dex-Induced Muscle Atrophy In Vivo

**Figure 1:**
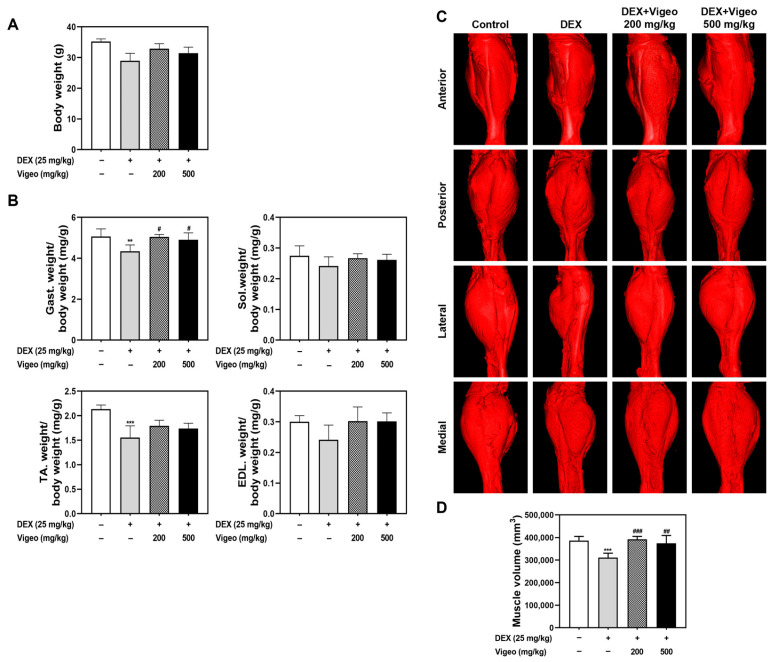
Effects of Vigeo on calf muscle properties in Dex-induced muscle atrophy in mice. (**A**) Mice with changes in body weight in each group with Dex-induced muscle atrophy. (**B**) Quantification of gastrocnemius (GA), soleus (SOL), tibialis anterior (TA), and extensor digitorum longus (EDL) muscle wet weight per body weight. (**C**) Radiological images of the calf muscles in four orientations: anterior, posterior, lateral, and medial. (**D**) Quantification of muscle volume in each group. *n* = 5 per group. Statistical analysis was performed using a one-way ANOVA test, the values are expressed as the mean ± SD of five mice. ** *p* < 0.01, *** *p* < 0.001 vs. normal control; # *p* < 0.05, ## *p* < 0.01, ### *p* < 0.001 as compared with Dex control.

According to several in vivo studies, dexamethasone, a synthetic glucocorticoid (GC), can induce muscle atrophy which reduces muscle mass, strength, and function [10,11]. Muscle disease progresses owing to the inhibition of protein synthesis and stimulation of protein degradation in skeletal muscle cells [5]. To determine whether Vigeo affects muscle atrophy in vivo, we used a Dex-induced muscle atrophy model with concomitant administration of Vigeo or DW in mice. The body weight of the Dex-treated mice was significantly lower than that of the control mice, suggesting that atrophy was normally induced by Dex treatment. The addition of Vigeo after Dex treatment tended to increase the body weight compared to that in the Dex-only group, suggesting the possibility that Dex-induced muscle atrophy could be improved by Vigeo treatment (Figure 1A). Additionally, to investigate the protective effects of Vigeo against Dex-induced muscle atrophy, we confirmed the properties of the gastrocnemius (GA), soleus (SOL), tibialis anterior (TA), and extensor digitorum longus (EDL) muscles in Dex-induced atrophy model mice using mCT imaging analysis. According to the mCT analysis method for leg muscle, the total muscle volume parameter and mCT images obtained using mCT analysis showed a prominent decrease in the Dex-injected mice compared to the control group (19.6% decrease; Figure 1D); however, it significantly increased in both the Vigeo low (250 mg/kg) and high (500 mg/kg) groups (26% and 20% increase for low and high doses, respectively; Figure 1C,D) against the Dex-injected group. Similarly, the weight of the GA muscle per body weight in the Dex-treated group was lower than that in the control group. Conversely, the GA muscle weights per body weight of the Dex + low Vigeo-supplemented group and the Dex + high Vigeo-supplemented group significantly recovered the muscle weight of the Dex-treated group. Weight gain in the SOL, TA, and EDL muscles per body weight also showed a similar weight change pattern in the Dex + Vigeo low and high dose treatments although not significantly different (Figure 1B).

### 3.2. Administration of Vigeo Protects Dex-Induced GA Muscle Damage In Vivo

**Figure 2:**
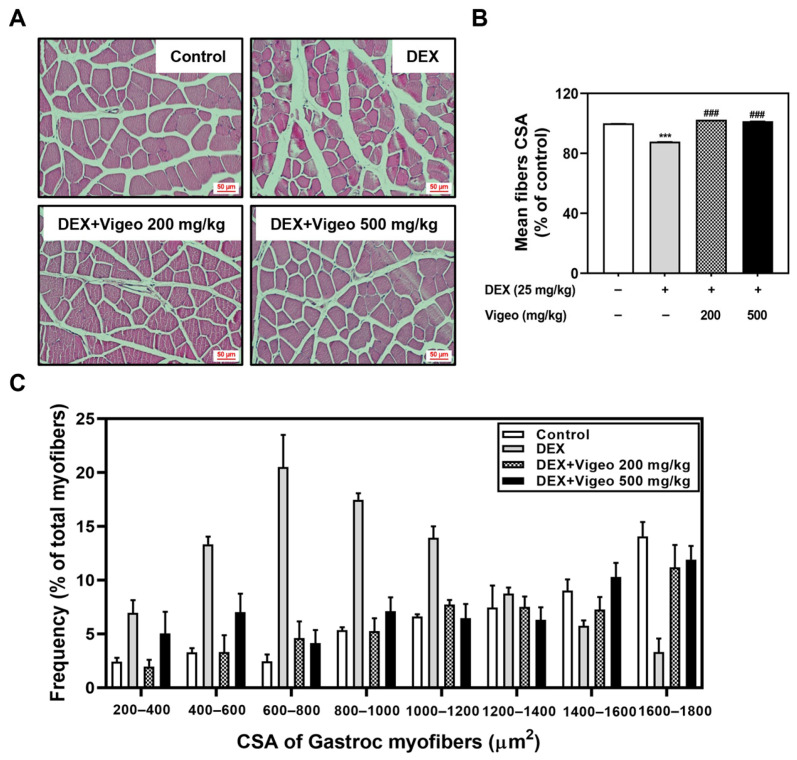
Effect of Vigeo on the muscle fiber cross-sectional area (CSA). (**A**) Representative hematoxylin and eosin (H&E) staining of GA muscle in each group. (**B**) Quantification of CSA of mice calf muscle is expressed as a relative percentage compared with control area. Quantification of muscle volume in each group. *n* = 5 per group. The values are expressed as the mean ± SD of five mice. *** *p* < 0.001 vs. normal control.; ### *p* <0.001 as compared with Dex control. (**C**) Distribution of the muscle fibers according to the CSA ranges (200–1800 μm^2^).

To confirm the protective effect of Vigeo against Dex-induced muscle atrophy, we performed a histological analysis of GA muscles. Dex treatment suppresses protein synthesis and promotes proteolysis [15,16]. Muscle atrophy induced by Dex results in a decrease in muscle fibers and their area. As shown in Figure 2A, muscle fibers and the CSA of muscle fibers in the Dex-induced group decreased compared to those in the control group, and increased in the Dex + Vigeo groups (200 and 500 mg/kg) (Figure 2A,B). Specifically, the distribution of muscle fiber diameter showed that the number of small muscle fiber areas (CSA range: 200–1400 μm^2^) increased in the Dex group compared to that in the control group. In contrast, the Dex group showed a significantly decreased distribution tendency in the large muscle fiber area (CSA range: 1400–1800 μm^2^) compared to the control group. However, Vigeo produced a similar muscle distribution pattern to the control group, with an improved number of large muscle fiber regions (Figure 2C).

### 3.3. Vigeo Suppresses the Protein Degradation Pathway Stimulated by Dex In Vivo

**Figure 3:**
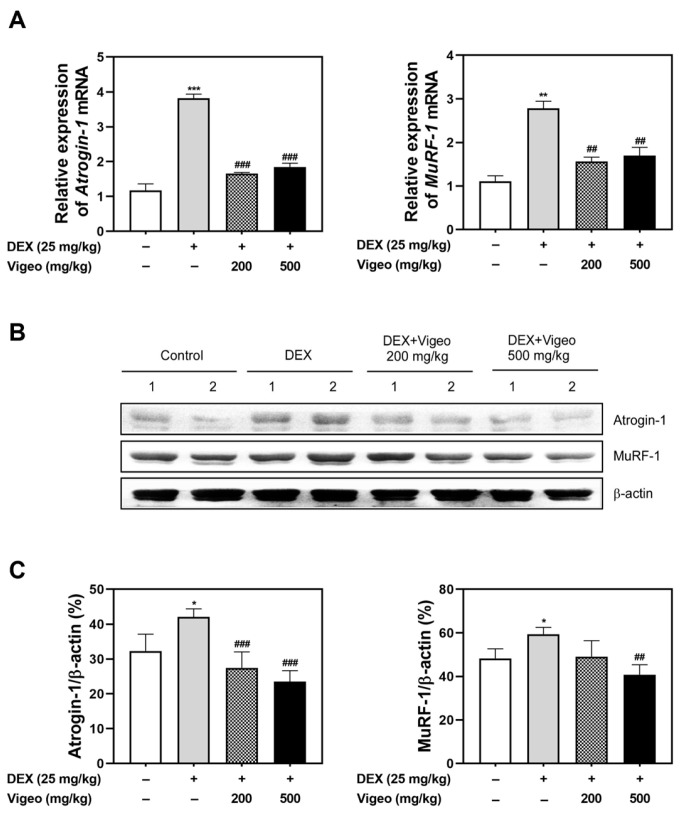
Effect of Vigeo on mRNA and protein expression levels of muscle atrophy markers in mice GA muscle tissues. (**A**) The mRNA expression levels of *atrogin-1* and *MuRF-1* were analyzed by quantitative real-time RT-PCR in GA muscle from each group. (**B**) The protein expression level of atrogin-1 and MuRF-1 was also confirmed with western blot analysis. Lanes 1 and 2 are the two randomly selected samples of each group. (**C**) The relative protein band intensity is represented as a relative percentage against the β-actin control blot. Data are expressed as the mean ± SD. * *p* < 0.05, ** *p* < 0.01, *** *p* < 0.001 vs. Normal control; ## *p* < 0.01, ### *p* < 0.001 as compared with Dex control.

Dex treatment leads to muscle atrophy, which is achieved through the process of a protein degradation system [15]. To understand how Vigeo affects the molecular signaling mechanisms underlying Dex-induced muscle atrophy, we altered the RNA and protein expression levels of the ubiquitin E3 ligases atrogin-1 and MuRF-1, which are markers of muscle proteolysis. We measured mRNA and protein levels in the GA muscles. Dex markedly increased the mRNA expression of proteolytic markers, including atrogin-1 and MuRF-1, compared to the control group. These increases showed that mRNA expression was effectively reversed by low and high doses of Vigeo (Figure 3A). Protein expression also showed an increase in the expression of Dex-induced proteolysis marker proteins; however, the expression was diminished in the Dex + Vigeo low and Dex + Vigeo high groups (Figure 3B). The pattern of change in protein expression is presented in the band quantification graph (Figure 3C). These findings suggest that Vigeo can improve muscle mass by regulating the degradation pathways during muscle atrophy.

### 3.4. Vigeo Improves Myoblast Differentiation by Stimulating the Expression of Myogenic Marker Genes in C2C12 Myoblast Cell In Vitro

**Figure 4:**
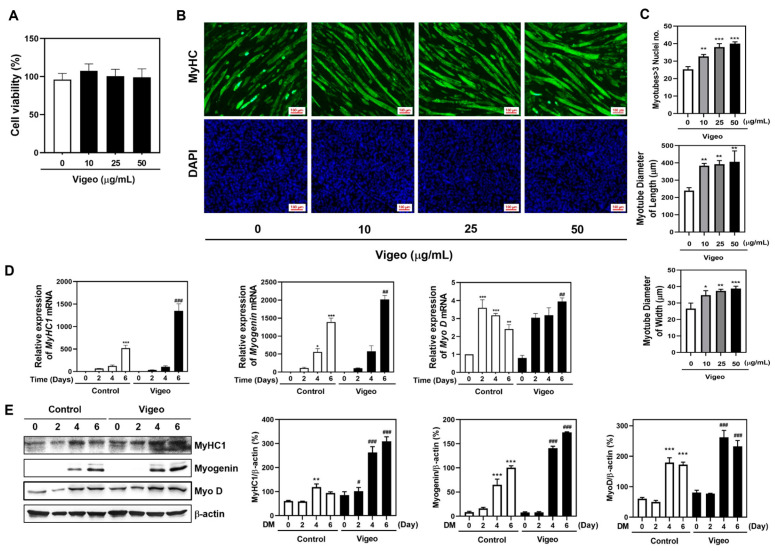
Effect of Vigeo on the expression of the MyHC and the formation of multi-nucleated myofibers in C2C12 myotube differentiation. (**A**) C2C12 cells were incubated with the indicated concentration of Vigeo (0, 10, 25, and 50 μg/mL). The cytotoxicity of Vigeo in C2C12 was analyzed by XTT assay. C2C12 myoblasts were cultured in the presence or absence of Vigeo 50 μg/mL with DM media for 5 d and followed by treatment with Dex 100 μM for 48 h. (**B**) Immunofluorescence staining of myotubes for MyHC (green) and DAPI (blue) was confirmed. (**C**) The graph presented shows the numbers of myotubes with three or more nuclei and the comparison of diameter for width and length of myotubes among the four groups. (**D**) Quantitative real-time RT-PCR and (**E**) western blot analysis of myoblast differentiation markers containing MyHc1, MyoD, and myogenin. Data are expressed as the mean ± SD. * *p* < 0.05, ** *p* < 0.01, *** *p* < 0.001 vs. Normal control; # *p* < 0.05, ## *p* < 0.01, ### *p* < 0.001 as compared with control at the indicated time (*n* = 3).

Based on the effects of Vigeo on Dex-induced muscle atrophy in in vivo models, as shown in Figure 1, Figure 2 and Figure 3, we validated how Vigeo fundamentally affects myoblast differentiation in the C2C12 culture system in vitro. Before myoblast differentiation, a toxicity test was conducted to assess the effects of Vigeo on myoblast growth. C2C12 cells were cultured in growth medium containing various concentrations of Vigeo (0, 10, 25, and 50 μg/mL) for 5 days. No significant differences in cell proliferation were observed after Vigeo treatment during the growing process (Figure 4A). To further investigate the influence of Vigeo on myoblast differentiation, C2C12 cells were cultured in DM supplemented with various concentrations of Vigeo (0, 10, 25, or 50 μg/mL) for 5 days. To evaluate differences in myotube shape, length, and width, we performed immunofluorescence staining and subsequent measurements. Vigeo increased the number of fused giant myotubes (three or more nuclei) in a dose-dependent manner (Figure 4B,C). Furthermore, Vigeo increased myotube diameter in terms of length and width in a dose-dependent manner compared to the control group (Figure 4B,C). We investigated the effect of Vigeo on the expression of marker genes that regulate myotube formation at each stage of myoblast differentiation. C2C12 cells were cultured in GM for 3 d, followed by each 2, 4, and 6-d incubation in DM in the presence of 50 μg/mL concentrations of Vigeo. Myoblasts exhibited a marked increase in mRNA and protein expression levels of MyHC1, MyoD, and myogenin during myogenic differentiation. The mRNA and protein expression of myogenic marker genes, including MyHC1, myogenin, and MyoD, significantly increased compared to those in the control group from DM4 to DM6 day with the addition of Vigeo (Figure 4D,E). These results indicated that treatment with Vigeo improved the expression of markers and promoted an increase in the size and number of myotubes.

### 3.5. Vigeo Up-Regulates Myoblast Differentiation by Suppressing Proteolysis Markers

**Figure 5:**
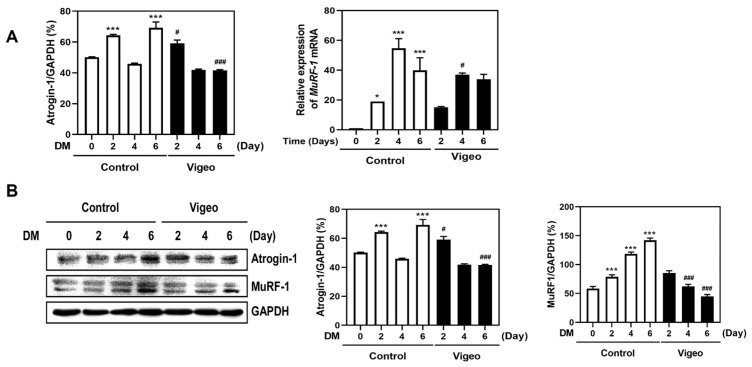
Effect of Vigeo on the myoblast differentiation and myotube atrophy in C2C12 cells. C2C12 cells were cultured with or without Vigeo (25 μg/mL) for indicated times (0, 2, 4, 6 days) in DM. (**A**) Quantitative real-time RT-PCR and (**B**) western blot were used for the analysis of mRNA and protein expression levels against myoblast differentiation markers including MyHC1, myogenin, and MyoD. Data represent means ± SD (*n* = 3). * *p* < 0.05, *** *p* < 0.001 vs. control at 0 day. # *p* < 0.05, ### *p* < 0.001 as compared with control at the indicated time.

Muscle atrophy is induced by an imbalance in protein lysis and degradation and is characterized by an increased level of protein degradation. Muscle breakdown mainly involves protein degradation via the ubiquitin-proteasome pathway in muscle cells [4,5]. Atrogin-1 and MuRF-1 are muscle-specific ubiquitin ligases and muscle atrophy markers [16]. Further, we determined whether the increase in the expression of these differentiation markers was due to the effect of Vigeo on protein degradation markers. The degradation markers atrogin-1 and MuRF-1, which increased during myoblast differentiation, were significantly decreased in mRNA and protein expression upon the addition of Vigeo (Figure 5A,B). Collectively, these results indicated that Vigeo promoted myoblast differentiation by inhibiting the expression of muscle-specific ubiquitin ligase markers.

### 3.6. Vigeo Attenuates Dex-Induced Muscle Atrophy through AMPK/Sirt-1/PGC1α and Akt/mTOR Signaling in C2C12 Myoblast Cells in Vitro

**Figure 6:**
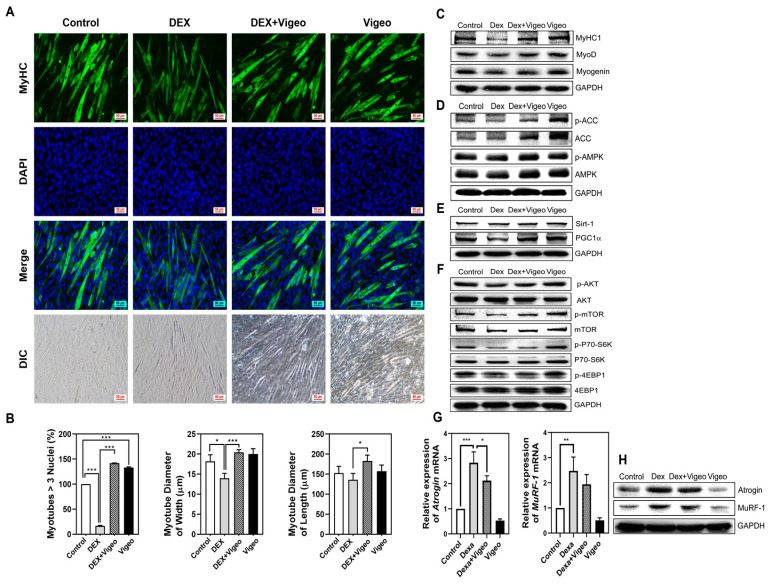
Effect of Vigeo on Dex-induced myotubes atrophy in C2C12 cells. The effect of Vigeo on myotubes degradation during myoblast differentiation was confirmed by (**A**) immunofluorescence staining of myotubes for MyHC (green) and DAPI (blue). (**B**) The graph presented shows the numbers of myotubes with three or more nuclei and the comparison of diameter for width and length of myotubes among the four groups. Data represent means ± SD (*n* = 3). * *p* < 0.05, *** *p* < 0.001 as compared with the indicated group. (**C**) Protein levels of MyHc1, MyoD, and myogenin were analyzed. (**D**) Protein levels of ACC and AMPK were analyzed. (**E**) Protein levels of Sirt-1 and PGC1α were analyzed. (**F**) Protein levels of AKT, mTOR, P70S6K, and 4EBP1 were analyzed. (**G**) The mRNA expression levels of *atrogin-1* and *MuRF-1* were analyzed by quantitative real-time RT-PCR in each group. Data represent means ± SD (*n* = 3). * *p* < 0.05, ** *p* < 0.01, *** *p* < 0.001 as compared with the indicated group. (**H**) Protein levels of atrogin-1 and MuRF-1 were analyzed.

Based on the in vivo experimental data showing that Vigeo has a positive effect on Dex-induced muscle atrophy, we examined whether the same tendency was observed in C2C12 cells in vitro. C2C12 cells were treated and cultured with Vigeo (0, 10, 25, and 50 μg/mL) at several concentrations for 5 d in DM. No significant differences were observed in cell proliferation in the presence of Vigeo compared to the vehicle in XTT cell proliferation analysis (Figure 4A). Further, we examined the effects of Vigeo on Dex-induced myotube atrophy in C2C12 cells. C2C12 cells were differentiated from myoblasts into myotubes in 2% HS DM media incubation for 5 d and followed further incubation with Dex treatment of 100 μM for 48 h. After treatment with Dex, we observed and analyzed the fusion index of differentiated myotubes under the conditions indicated in Figure 6A. The elongated myotubes were represented by MyHC green-immunofluorescence-stained cells and blue-stained DAPI in the elongated myotubes showed as fused myotubes; only myotubes formed by the fusion of three or more nuclei were counted. Dex treatment resulted in severely atrophied myotube formation, which was distinct from the thick, well-formed myotubes observed in control cells. Contrary to the Dex-only treated group, the cells treated with Dex + Vigeo (50 μg/kg) showed improved myotube differentiation, resulting in the recovery of the width and length of the myotubes (Figure 6A,B). To compare the protective effects of Vigeo against Dex-induced muscle cell atrophy, we verified the expression of genes related to myoblast differentiation, mitochondrial function, protein synthesis, energy metabolism, and muscle cell degradation in C2C12 myotubes. Dex-treated cells showed remarkably reduced myoblast differentiation, which was confirmed to result in decreased expression of myoblast differentiation-related genes, such as MyHC1, MyoD, and myogenin. However, the expression of muscle cell differentiation factors decreased after Dex treatment, and the expression of differentiation markers recovered in response to Vigeo treatment (Figure 6C). In skeletal muscle cells, mitochondrial biogenesis is one of the important parts of maintaining healthy muscle cells, and among them, the PGC1α is a key regulator of mitochondrial biogenesis and function [17] and is commonly activated by AMPK and Sirt-1 [18,19]. As muscle cell differentiation progressed, the expression of genes involved in mitochondrial biogenesis increased, and the expression of AMPK, Sirt-1, and PGC1α decreased following Dex treatment. The inhibition of myoblast differentiation was expected to be due to a Dex-induced decrease in mitochondrial biogenesis and this decrease was reversed through increased expression of AMPK, Sirt-1, and PGC1, α, which was strongly restored by Vigeo treatment (Figure 6D–E). Skeletal muscle mass and differentiation are regulated by protein synthesis via the mTOR pathway [20]. To evaluate the effects of Vigeo on muscle protein synthesis in response to Dex stress, changes related to the Akt/mTOR pathway were measured in C2C12 cells. Dex treatment inhibited the phosphorylation of Akt and the mammalian target of rapamycin (mTOR) compared with the control. However, the Dex + Vigeo group showed a significant increase in Akt and mTOR phosphorylation (Figure 6F). To further determine the effect of Vigeo on muscle anabolic signaling downstream of Akt/mTOR signaling [21], the phosphorylation of 4EBP1 [22] and p70S6K [23] was detected in C2C12 myotubes. The Dex-treated group also showed that the phosphorylation of p70S6K and 4EBP1 was significantly decreased in C2C12 myotubes. However, the phosphorylation levels of p70S6K and 4EBP1 were slightly increased in the Dex + Vigeo group compared to those in the Dex group (Figure 6F). Finally, concurrent restraint of atrogin-1 and MuRF-1 resulted in myotube hypertrophy, which mainly reflects the upregulation of protein synthesis and prevents Dex-induced myotube atrophy [24]. We cultured myotubes to determine whether Dex-induced muscle loss was affected by the myoblast lytic mechanism induced by the addition of Vigeo. In C2C12 cells, both mRNA and protein expression of the muscle proteolysis markers atrogin-1 and MuRF-1 were significantly increased compared to those in the control due to additional Dex treatment. However, the expression of these two muscle-specific degradation factors was significantly decreased by Vigeo treatment, indicating a tendency to compensate for muscle loss (Figure 6G,H). Together, these results demonstrate that Vigeo prevents muscle atrophy and promotes myoblast differentiation by suppressing Dex-induced proteolysis and regulating mitochondrial biogenesis and energy metabolism of muscle cells in C2C12 myotubes.

## 4. Discussion

A growing body of research attempts to treat or alleviate sarcopenia using natural products, synthetic drugs, and exercise to improve muscle strength and endurance. A promising approach to treat or prevent sarcopenia is the use of natural products derived from plants, animals, and minerals [25] which have been used for centuries to treat various medical conditions, including muscle loss. Particularly, natural products and herbal medicines have been used around the world for centuries to treat human disease and maintain health and are gaining increasing attention for their bioactivity, efficacy, and safety [26]. Natural fermented products have long been recognized as crucial targets for drug discovery and development, owing to their diverse and complex chemical structures and biological activities [27]. In addition, mixed fermentation extracts can increase the yield of known and novel metabolites and induce the formation of analogs of known metabolites [28]. Vigeo is a fermented combined extract of ESM, AJN, and AJK using the traditional Korean nuruk fermentation method. ESM, a traditional medicine used in Korea, Japan, and China as a treatment for pain control and improvement of functional disability in patients with osteoarthritis [29], has been reported to protect against bone damage and exert anti-osteonecrosis effects in vitro and in vivo [30,31]. AJN is a herbal medicine traditionally used to treat obesity and its associated complications [32] and has been reported to have anti-inflammatory [33], anti-arthritic [34], and antioxidant [35] properties. AJK also exhibits a variety of bioactive properties, including anti-inflammatory [36], antioxidant [37], and anti-obesity activity [38]. In our previous study in 2021, Vigeo was used as a fermented extract produced by fermenting a starter called Korean nuruk with yeast, rice, and a mixture of three dried plants (ESM, AJN, and AJK) under standardized conditions. We clarified that the fermented extract Vigeo has the potential for anti-osteoporotic activity by inhibiting the phosphorylation of MAPKs (p38, ERK, and JNK), IκB, and AKT in early signaling of osteoclast precursor cells and suppressing the expression of mRNA and protein of c-Fos and NFATc1 transcription factors. Moreover, the bone-resorption ability of mature osteoclasts generated by the co-culture of osteoblasts and bone marrow cells was significantly inhibited by Vigeo treatment of dentin slices. These in vitro findings showed that the administration of fermented Vigeo extract strongly prevented LPS-induced inflammatory osteoporotic bone loss in mice [12]. Our study also reports that Vigeo inhibited arthritis symptoms, including bone loss and serum levels of TNF-α, IL-6, and IL-1β, in a mouse model of collagen-induced arthritis/rheumatoid arthritis [39]. The present study is the first to identify the efficacy of Vigeo on muscle disease conditions caused by the inhibition of muscle cell differentiation and muscle atrophy in vitro and in vivo. The main bioactive components of ESM are known to be phenols and saponins [40], while AJN contains many effective components that inhibit osteoarthritis and inflammation, including inokosterone, oleanolic acid, saponin, and ecdysone [29]. AJK has also been found to have components with important functional therapeutic activities, including sesquiterpenes, atractylone, 3-β-hydroxyatratylon, selina-4, hinesol, and β-eudesmol and sesquiterpenoid glycosides [41]. The bioactivity of Vigeo may be induced by the main constituents of each of the three plants, but it may also be induced through secondary metabolites produced by nuruk fermentation. Therefore, further fermented extract analysis studies are needed to identify the specific bioactive molecules that affect the muscle metabolism of Vigeo.

In this study, Dex was used to induce muscle loss in in vitro and in vivo models, and the efficacy of the fermented extract of Vigeo in the treatment of Dex-induced muscle loss was evaluated. In vitro experiments showed that C2C12 myoblasts were stimulated with 2% horse serum to differentiate into green-MyHC-IF-stained mature myotubes containing multiple nuclei. However, the MyHC intensity of the strongly green-stained myotubes declined drastically after Dex treatment. The number of multi-nucleated myotubes and the length and thickness of myotubes were also reduced by Dex treatment. In contrast, in the group treated with both Dex and Vigeo, the formation of Dex-induced atrophied myotubes was significantly recovered despite Dex stress (Figure 6A,B). The improvement in the number of multinucleated myotubes in the Vigeo-treated group compared to that in the control group suggests that Vigeo promotes myoblast differentiation during myogenesis.

The formation of skeletal muscle cells is controlled by myogenic regulatory factors (MRFs), including Myf5, MyoD, myogenin, and MRF4 during postnatal myogenesis [42]. Myf5 and MyoD are the key transcription factors that play essential roles in skeletal muscle differentiation. MyoD, known as myoblast determination protein 1, is expressed in early myogenic cells and it regulates the cell cycle, activates transcription factors such as myogenin, and participates in the fusion of myogenic cells which is absolutely necessary to form multinucleated muscle fibers [43,44]. Myogenin is activated by MyoD and acts to downregulate Myf5 and upregulate MRF4 expression. Myogenin is essential for skeletal muscle differentiation, and myogenin-deficient mice are unable to form mature secondary skeletal muscle fibers [45,46]. In the present study, Vigeo treatment increased the mRNA and protein expression levels of MyoD and myogenin (Figure 4). These results suggest that Vigeo treatment effectively upregulates myoblast differentiation by stimulating the expression of myogenic marker genes, such as MyHC, MyoD, and myogenin.

Dex, well known as a synthetic glucocorticoid, induces muscle degradation, downregulates muscle differentiation, and causes muscle atrophy upon long-term administration or high-dose exposure. Dex treatment can cause muscle loss through decreasing protein synthesis and increasing ubiquitin-proteolysis systems consisting of atrogin-1 and MuRF-1 [47,48]. In this study, Vigeo treatment strongly inhibited the protein degradation system by suppressing the mRNA and protein expression of atrogin-1 and MuRF-1 in myoblast differentiation. Furthermore, as predicted, we found that Vigeo was significantly effective in the recovery of muscle loss induced by Dex both in vitro and in vivo (Figure 1, Figure 3 and Figure 6). In the mCT analysis, the gastrocnemius muscle mass that had decreased due to Dex stress was significantly restored to a level similar to that of the normal control, caused by suppression of the expression of degradation markers by Vigeo administration in vivo (Figure 1, Figure 2 and Figure 3). In vitro experiments also confirmed that the muscle degradation and loss improved by Dex as shown in vivo was noticeably restored to normal levels by the addition of Vigeo (Figure 6A,B). During myoblast differentiation, recovery of protein expression of myoblast differentiation markers, including MyHC1, MyoD, and myogenin, was observed (Figure 6C), and it was confirmed that the mRNA and protein expression of atrogin-1 and MuRF-1, which are muscle cell degradation markers increased by Dex treatment, was reduced by Vigeo administration (Figure 6G,H). To preserve healthy skeletal muscles, maintaining an appropriate balance between protein synthesis and degradation is necessary [49]. The AMPK/Sirt-1/PGC1α signaling pathway is crucial for mitochondrial biosynthesis and energy metabolism in skeletal muscle [50]. AMPK regulates the expression of Sirt-1, which leads to deacetylation of the downstream Sirt-1 target (PGC1α) [51]. In this study, the AMPK/Sirt-1/PGC1α signaling pathway, which was atrophied by Dex treatment in C2C12 cells, showed a significant increase in the expression of these three genes following Vigeo treatment. This improvement in the expression of mitochondrial biosynthesis markers suggests that Vigeo positively regulates mitochondrial biosynthesis in C2C12 myoblasts. The Akt/mTOR signaling pathway is crucial for maintaining the balance between protein biosynthesis and degradation. The activation of Akt leads to the phosphorylation and activation of downstream proteins, such as mTOR, p70S6K, and 4EBP1 [22,52]. However, activated Akt/mTOR signaling inactivates atrophy-induced genes, such as atrogin-1 and MuRF-1 in muscle cells [53]. In this study, the influence of Vigeo treatment on the phosphorylation levels of Akt, mTOR, p70S6K, and 4EBP1 was determined by the improved expression levels of protein synthesis markers compared to those in the Dex-treated control group in C2C12 myotube cells (Figure 6F), suggesting that changes in the expression of protein synthesis markers caused by Vigeo treatment might contribute to the decreased expression of protein degradation genes, including atrogin-1 and MuRF-1. Further studies in other muscle wasting models are needed to clearly understand the mechanism of action of Vigeo in pathological muscle atrophy models caused by multiple factors such as aging and energy and nutritional imbalances.

## 5. Conclusions

The present study demonstrated that the administration of fermented Vigeo extract has a therapeutic effect on Dex-induced mouse skeletal muscle atrophy in vivo and that the efficacy of Vigeo might be regulated by inhibiting the proteolytic degradation system through AMPK/Sirt-1/PGC1α and Akt/mTOR signaling in C2C12 myoblast cells in vitro. Finally, the fermented extract of Vigeo may be a suitable candidate for the treatment of sarcopenia accompanied by muscle atrophy and dysfunction.

## Data Availability

The data used to support the findings of this study are available from the corresponding author upon request.
